# One Health Approach to Identify Research Needs on *Rhipicephalus microplus* Ticks in the Americas

**DOI:** 10.3390/pathogens11101180

**Published:** 2022-10-13

**Authors:** Agustín Estrada-Peña, Alina Rodríguez Mallón, Sergio Bermúdez, José de la Fuente, Ana Domingos, Mario Pablo Estrada García, Marcelo B. Labruna, Octavio Merino, Juan Mosqueda, Santiago Nava, Ricardo Lleonart Cruz, Matías Szabó, Evelina Tarragona, José M. Venzal

**Affiliations:** 1 Department of Animal Health, Faculty of Veterinary Medicine, 50001 Zaragoza, Spain; 2 Research Group in Emerging Zoonoses, Instituto Agroalimentario de Aragón (IA2), 50013 Zaragoza, Spain; 3 Animal Biotechnology Department, Center for Genetic Engineering and Biotechnology, P.O. Box 6162, Havana 10600, Cuba; 4 Instituto Conmemorativo Gorgas de Estudios de la Salud, Panama 0801, Panama; 5 SaBio, Instituto de Investigación en Recursos Cinegéticos (IREC-CSIC-UCLM-JCCM), Ronda de Toledo s/n, 13005 Ciudad Real, Spain; 6 Department of Veterinary Pathobiology, Center for Veterinary Health Sciences, Oklahoma State University, Stillwater, OK 74078, USA; 7 Global Health and Tropical Medicine, Instituto de Higiene e Medicina Tropical, Universidade Nova de Lisboa (GHTM-IHMT-UNL), 1099-085 Lisbon, Portugal; 8 Center for Genetic Engineering and Biotechnology, P.O. Box 6162, Havana 10600, Cuba; 9 Faculty of Veterinary Medicine, University of Sao Paulo, Sao Paulo 05508-220, Brazil; 10 Faculty of Veterinary Medicine, University of Tamaulipas, Ciudad Victoria 87000, Mexico; 11 Immunology and Vaccines Laboratory, College of Natural Sciences, Autonomous University of Queretaro, Queretaro 76010, Mexico; 12 Instituto Nacional de Investigaciones Agropecuarias, Estación Experimental Agropecuaria Rafaela—Instituto de Investigación de la Cadena Láctea (INTA-Consejo de Investigaciones Científicas y Técnicas), Rafaela, Santa Fe 45890, Argentina; 13 Instituto de Investigaciones Científicas y Servicios de Alta Tecnología (INDICASAT AIP), Panama 0801, Panama; 14 Sistema Nacional de Investigación (SNI), Panama 0801, Panama; 15 Faculty of Veterinary Medicine, Federal University of Überlandia, Uberlandia 38408-100, Brazil; 16 Departamento de Parasitología, CENUR Litoral Norte, Universidad de la República, Salto 50000, Uruguay

**Keywords:** *Rhipicephalus microplus*, ecology, climate, pasture vacancy, integrated control, One Health, research agenda

## Abstract

We aim to provide a harmonized view of the factors that affect the survival and promote the spread of *R. microplus* in the Neotropics, approaching its different facets of biology, ecology, distribution, and control. We review the interactions among environmental niche, landscape fragmentation, vegetal coverage (abiotic traits), and the biotic aspects of its ecology (abundance of domesticated or wild competent hosts), proposing emerging areas of research. We emphasize a holistic view integrating an economically and ecologically sustainable control of infestations and transmitted pathogens by *R. microplus* in the Neotropics. Examples of research link the trends of climate, the composition of the community of hosts, the landscape features, and a tailored management based on ecological grounds. Our view is that factors driving the spread of *R. microplus* are complex and deeply interrelated, something that has been seldom considered in control strategies. The effects of climate may affect the dynamics of wildlife or the landscape composition, promoting new patterns of seasonal activity of the tick, or its spread into currently free areas. In this paper we encourage a One Health approach highlighting the main aspects governing the components of the tick’s life cycle and its interactions with livestock and wild animals.

## 1. Introduction

The affections to animal health by ticks and transmitted micro-organisms in the Neotropical region are numerous. *Rhipicephalus microplus* (the cattle tick) is probably the most important pest to cattle in the region, because its direct effects on the production via blood intake and side effects, costs related to control, and its ability to transmit pathogens such as *Babesia* *bovis*, *B. bigemina* or *Anaplasma* *marginale*, are responsible for massive economical losses [1]. Once imported from yet unknown origin and destination points, it now covers a large territory from some areas of Southern USA and Mexico to parts of northern Argentina and Uruguay. The tick has a one-host short life cycle, therefore promoting from three to up to five or six generations per year according to climatic conditions, increasing the needs of control measures throughout the year. Economical losses are increased by an uncoordinated chemical control of which no significant advances have occurred in the last 15–20 years. Not only the contamination of the ecosystems and the presence of residues in meat or milk using chemicals is a reason for concern, but also the development of resistant strains of the tick, as consequence of the use of synthetic chemicals as the principal tool for cattle tick control, are caveats that should be minimized by incorporating management strategies as complement to the use of a particular tool as the synthetic chemical acaricides. These alternatives include pasture spelling methods based on tick-ecology knowledge, the use of tick resistant breeds of cattle, and the incorporation in an integrated management program of tick-vaccines when options with adequate efficacy are commercially available [2,3,4].

Decision support systems could be used to manage the seasonal forecasting of the activity and abundance of *R. microplus* [5]. However, this approach is unreliable without the understanding of the ecological factors affecting the control or eradication of the cattle tick. Developing improved decision support systems becomes more important as the demand for sustainable cattle production is growing and pest pressure increases under climate change. *Rhipicephalus microplus* has a pantropical distribution. A sustainable and cost-effective control of the cattle tick in central and south America is still far to be achieved. Its possible spread throughout the target area by the climate trends or the deforestation practices favoring cattle farming has been only partially studied [6]. An approach for the control of *R. microplus* should ideally consider, address, and integrate (i) an accurate knowledge of the population dynamics of *R. microplus* in a particular region; (ii) the status of resistance to different molecules used as acaricides; (iii) forage availability in order to incorporate pasture spelling options; (iv) issues regarding public health (i.e., accumulation of chemical residues in meat or milk by using acaricides with long a withdrawal period); (v) the adoption of more resistant strains of cattle to ticks and/or an adequate vaccine program; (vi) the climate, its trends, and ecosystem health; (vii) the use of soil; (viii) the presence and abundance of wild ungulates in the specific sites where it becomes relevant; and (viiii) the use of live vaccines against cattle babesiosis and anaplasmosis once they are available and officially approved would contribute to reducing the number and frequency of acaricide treatments against the tick vector.

The main aim of this paper is to revisit the different facets affecting the biology, ecology, distribution, and abundance of *R. microplus*, focusing on their interactions, and proposing emerging areas of research. We aim to provide an integrative view of the factors affecting the survival and promoting the spread of *R. microplus* in the target region, looking for an integrated, economically and ecologically sustainable control of infestations by *R. microplus* on cattle. We would like to encourage in the following lines a One Health approach highlighting the main aspects governing the components of the tick’s life cycle.

## 2. The Abiotic Niche (Weather and Climate)

**Figure 1:**
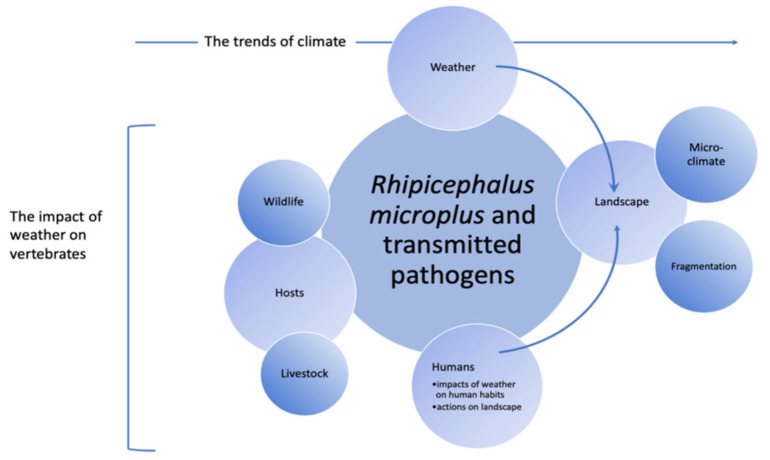
A synoptic view of the abiotic and biotic factors affecting the survival, development, and control of *R. microplus* in the Americas.

Figure 1 summarizes the main factors that could affect the survival and activity of *R. microplus*. The abiotic niche is probably on the top of the pyramid of traits affecting the survival and spreadability of a tick. Abiotic niches include the temperature, humidity, water vapor deficit, or other atmospheric variables, known as macroclimate, which regulate both the survival and the development rates of a tick. Microclimate and landscape features can also be considered as abiotic traits [6]. *Rhipicephalus microplus* has only two off-host stages, namely the developing eggs, and the questing larvae, therefore reducing the chances that trends of macroclimate to be the only responsible of an impact on the distribution of *R. microplus*. There is a substantial body of studies in the Neotropics on the dynamics of both parasitic and non-parasitic phases at different latitudes and how they determine the number of annual generations according to climate and soil use conditions [2,7,8,9,10,11,12,13,14,15,16,17,18,19,20,21,22,23,24,25,26]. In subtropical latitudes, it is estimated that a shorter winter would promote an earlier beginning of activity the next spring and, therefore, a variable number of generations, providing that suitable soil/atmospheric water contents are available. An extra number of generations, driven by shorter winter and warmer weather conditions, would lead to extra costs in cattle production, originated by both the parasite-derived losses and the acaricide treatments (along with the possibility of increasing the development of resistance). It has been demonstrated that a warmer niche may contribute to the spread of *R. microplus* further south in Argentina if the humidity remains within the range allowing the survival of the tick [6,22]. Warmer climates may also be responsible for the spread of *R. microplus* to higher altitudes, such as those over 2600 m a.s.l., in Colombia [26].

With an off-host shorter life cycle, the number of generations of the tick taking a blood meal on bovine hosts would be higher. However, this is a topic that requires additional consideration, because a warmer climate does not automatically translate into a better environment for cattle management [27]. The use of weather variables for capturing the probable distribution of a parasite is intended for an indication of its potential distribution [28]. However, a tick needs to feed on hosts (domestic cattle being the most important in the target region), and it is therefore necessary to evaluate whether cattle farming could persist in these newly available areas for the tick. In this sense, it has been concluded that models based solely on climate data, disregarding how the land use affects the presence of cattle and the viability of the non-parasitic phases of *R. microplus*, could overestimate the potential range of a tick [22].

Temperature might affect the development of pathogens in the tick. It has been observed, at least for *B. bovis,* that infection rates in the tick increase when ticks are exposed to lower temperatures (14 °C) than to higher temperatures (27 °C) [29]. However, if infected larvae are heated at 37 °C for 3–5 days, they can infect cattle without the required 3–5 days of feeding [30]. For *B. bigemina,* it is known that temperature affects transovarial infection rates, with the highest infection in eggs being between 26 °C and 30 °C [31]. These studies were conducted a long time ago in laboratory conditions. Unfortunately, studies on the climate change, the pathogen transmission and its impact on animal health have been neglected.

Basic modelling of the expected distribution of *R. microplus* in the Nearctic-Neotropics was first done in 1999 [32]. Efforts to discern how the climate trends and land use were involved in the spread of the tick were later addressed [6,22,33]. However, these studies did not include the impact of a changing climate on the potential hosts for the tick, which are necessary for the establishment of permanent populations (see below). Other models improved the assessment of the mortality and development rates of *R. microplus*, providing an estimation of the seasonality of the tick populations, and therefore a tool to predict the “waves” of questing larvae [34].

The ecological studies of the off-host dynamics of *R. microplus* provide a significant and solid framework for the application of pasture vacancy as a tool for tick control as complement to the chemical control. In fact, pasture spelling is a feasible alternative to minimizing the annual frequency of acaricides applications. Pasture spelling controls ticks by denying host access to free-living larvae that will then die from starvation and desiccation. The determination of the spelling periods required to achieve significant control of *R. microplus* in different periods of the year at a particular area is the basic knowledge necessary to apply this method. In this sense, there are studies in the Neotropics that already addressed this subject and raised proposals on the adequate application of these methods at different latitudes [8,19,21,35,36,37]. Additionally, some models have been developed to infer the impact of different management methods to achieve a maximum mortality of questing larvae in the grass, thus “cleaning” the pasture [38,39]. However, pasture spelling has drawbacks because of differences in the use of food resources. Thus, it is convenient to incorporate knowledge about forage availability before the application of modeling methods. In addition, it must be kept in mind that pasture vacancy means additional costs to feed cattle, which is unbearable for small producers, and it should be kept to periods as short as possible to be cost effective.

**Figure 2:**
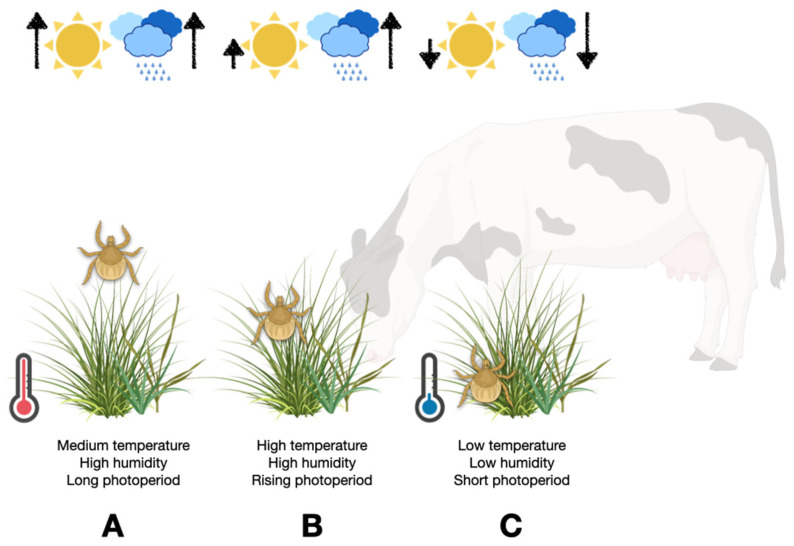
The relationships between macro- and microclimate features affecting the development of eggs, survival, and activity of the questing ticks. For an optimal questing, ticks must climb on top of the vegetation; however, keeping this position for a long time produces losses of water, pushing the tick to the base of the vegetation to retake water vapor and rehydrate. A wet climate will enlarge the questing periods of the tick, resulting in a higher risk for grazing cattle. Also, wet conditions improve the survival of the ticks. The figure schematizes three different conditions that occur in parts of the Neotropics, regarding the photoperiod (short or long), the temperature (warm or cold) and the rainfall-relative humidity (high or low) and how they affect the quiescence or questing of larvae within the vegetation.

The models already developed for the life cycle of *R. microplus* estimate either its expected range under various sets of environmental conditions or to provide an estimation of its mortality and/or duration of the incubation. Details on seasonality have been indirectly obtained from these models (i.e., 5, 34, 38–39). A meta-study of the modelling approaches developed for boophilid ticks is available [40]. There are, however, additional approaches called physiological models which were developed for epidemiological studies involving insects like mosquitoes [41,42]. A physiological model estimates the energy consumption of the target organism across a range of weather conditions. It is thus feasible to gain knowledge on the true impact of a gradient of weather variables to finely tune the modeling of a species. As mentioned, we are aware of only one similar approach [38] that has been used for further improvements of models for *R. microplus*. Figure 2 synthetizes how some microclimate features may affect the questing of the larvae of *R. microplus*.

## 3. The Wildlife as Competent Hosts of *R. microplus*

**Figure 3:**
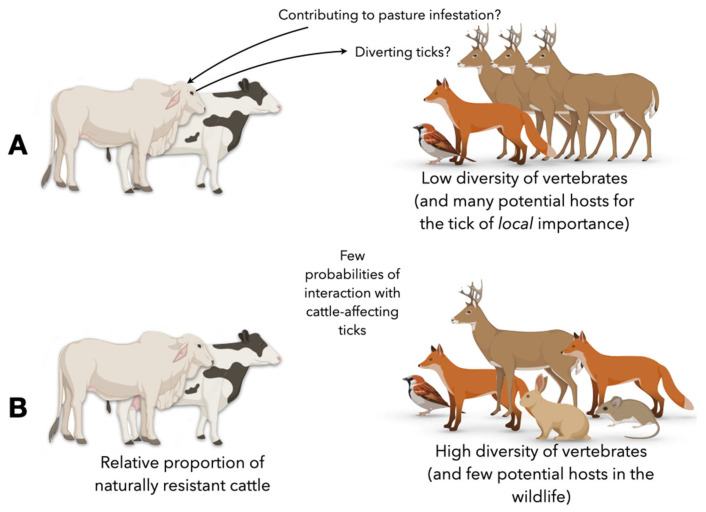
The concept of the heterogeneity of the communities of wild vertebrates influencing the prevalence of *R. microplus* on domestic cattle. (**A**) Under local conditions a low variability of the community, in which wild ungulates are prevalent and may act as hosts of the tick, may distract ticks from the cattle farming areas and also contribute to keeping a permanent population of ticks. (**B**) a high variability of the community, with few alternative hosts, may promote a lower pressure of ticks because they contribute little to the feeding of the tick. Of note, there is compelling evidence that livestock is the most important (or most of the times, the only) host for *R. microplus* but under some circumstances, wild ungulates may have a role for which adequate information does not exist. We claim the need to observe in the field the composition of the wildlife communities, the movements of the different species across the landscape, and their contribution to the feeding of the tick.

It was taken for granted that cattle and other livestock, like horses and donkeys, were the only hosts for *R. microplus*, but studies have demonstrated that other native or introduced ungulates may feed this tick [43]. The white-tailed deer, *Odocoileus virginianus*, has been repeatedly reported as an important host for the cattle tick, at least in large areas of the USA and Mexico [44], and the history of its involvement in the eradication campaign has been addressed [43] (Figure 3).

However, this is not the only wild host for the tick, since up to 40 species of mammals and birds from which bona fide records of the cattle tick exist, although there is no information on the competence of most of them to sustain tick development and reproduction [45]. In other words, domestic cattle are the most important hosts for *R. microplus* (and most of the times the only one) in the target region. Under special circumstances and for some sites, wild ungulates may take a secondary role keeping permanent populations of the tick or by introducing *R. microplus* in tick free areas with suitable ecological conditions for the tick. The tick has been found on up to six species of wild Cervidae with different ranges and habits. For example, it has been reported that both *O. virginianus* and *Mazama temana* may be hosts for the cattle tick in Mexico [46], with a prevalence of 28% and an intensity of 25.2 ticks/animal. The density of wildlife in some grazing areas may contribute to the support of large populations of *R. microplus*, even if an adequate anti-parasitic management of livestock is carried out [47,48].

*Rhipicephalus microplus* was observed to parasitize the Marsh deer (*Blastocerus dichotomus*) under natural conditions with an increasing prevalence (up to 100%) under conditions of natural habitat degradation in Brazil [49], and it was demonstrated that captive Marsh deer may sustain cattle tick populations on its own [50]. In Brazil, the pampas deer, *Ozotoceros bezoarticus*, showed higher values of abundance, intensity, and prevalence of *R. microplus* than cattle. It also demonstrated the ability to maintain populations of this tick [51]. Molecular studies indicate that *O. bezoarticus* is coinfected by different hemoparasites, including *B. bovis*, *B. bigemina*, and *A. marginale* [52]. In tropical areas of the American continent, water buffalo (*Bubalus bubalis*) has become established as a species raised and bred for its meat and milk but especially for its resistance to parasites and humid conditions. Although water buffaloes could sustain *R. microplus* populations, they have a higher resistance to *R. microplus* infestation than that of the bovine host [53]. Thus, buffaloes could help to reduce the frequency of annual acaricide treatments if they are bred in rotational regimes of paddocks also grazed by cattle.

Communities of vertebrates are taxonomic assemblages at the large spatial scale. A community thus represents the combination and relative proportions of vertebrate species (hosts and non-hosts for the tick) occurring in a territory. Such relative composition depends on landscape features promoting adequate food and shelter, predators and/or competitors, and the climate preferences of each species. The wild ungulates may be dominant in some communities; in others their presence may be purely testimonial. Both conditions may have different impacts, not yet quantified, on the spread of populations of *R. microplus* in sites where domestic cattle is absent, or diverting ticks form the livestock. The point is that either climate trends or the promotion of farming for hunting species of introduced ungulates could promote changes in the composition of communities that share the habitat with *R. microplus* [6]. It has been demonstrated that these ungulates commonly used in the southern USA for hunting can support permanent populations of *R. microplus* [47,48,54]. Further research on the role of exotic wild ungulates in the spread of *R. microplus* ticks at local scale are clearly needed.

## 4. The Landscape and Its Impact on *R. microplus*

The term “landscape” has been classically used in the scientific literature with two different meanings when applied to the ecology of ticks. One of these connotations refers to the vegetal species composition of a target region. The vegetal cover may influence the prevailing climate, resulting in what is known as the microclimate (Figure 3); such a microclimate is the set of environmental conditions that provide the niche for *R. microplus* for female oviposition, egg hatching, and larval questing. To note, vegetal composition may also affect the cattle stocking rates of the site or the attractiveness of the site to wild ungulates, both influencing the abundance of ticks in the site. The second meaning of “landscape” refers to the disposition of the mosaic of habitat/non habitat for the tick, mostly referring to individual patches favoring tick survival and questing. The patches of suitable habitat may be linked by “corridors” or preferred routes of movements of the herds of either livestock or wildlife. In any case, the landscape is affected by the dominant climate and the human actions on the vegetal cover (i.e., the massive deforestation for farming). In this sense, the suitability of areas for the permanent establishment of *R. microplus* considering both the climatic and soil suitability for the ticks and the availability of grazing cattle have been analyzed and modeled [21,33]. The size and distance among landscape patches and the existence of “preferred” corridors for hosts were demonstrated to influence the density of ticks by comparing a model of the landscape traits with actual questing densities obtained from field surveys of the European tick *Ixodes ricinus* [55,56]. The concept, incorporating tools such as cellular automata, was applied to the probability of importation of *R. microplus* into the southern USA by the white-tailed deer [57]. Such earlier studies demonstrated that the sites with high probability of breaking the quarantine line in southern Texas have a mathematical definition given by the relationships between the spatial arrangement of the landscape patches and the movements of the vertebrates throughout. These results were supported soon after by actual records of *R. microplus* in Zapata Co., Star Co. or Webb Co. in southern Texas [47]. We would like to encourage further efforts on similar field studies focused on *R. microplus*, since those are of pivotal importance for a sustainable and economically adequate tick control.

**Figure 4:**
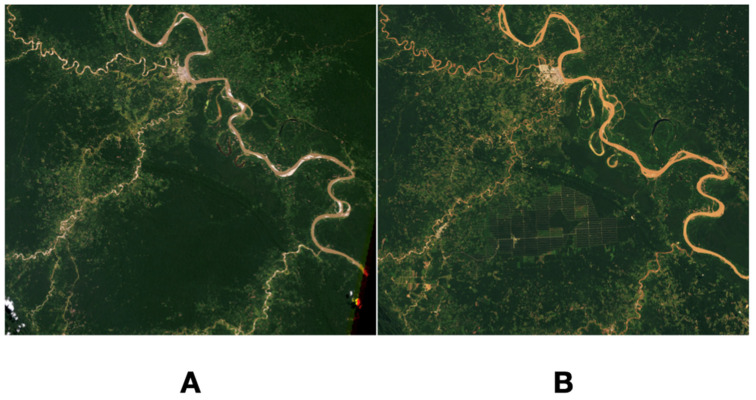
Two actual examples of the deforestation rate of the Amazonian rain forest in Brazil, as captured by satellite images. (**A**) the unaltered rain forest, around the year 2000. (**B**) the deforested rain forest in the same area as before, around the year 2010, showing in the middle of the image large areas without the natural forest, and devoted to grazing, soybean, and sugar cane cultures. Original NASA Earth Observatory images prepared by Lauren Dauphin, using Landsat data from the U.S. Geological Survey (available at http://earthexplorer.usgs.gov/ (accessed on 15 September 2022)), MODIS data from NASA EOSDIS/LANCE and GIBS/Worldview (available https://earthdata.nasa.gov/ (accessed on 15 September 2022)) and forest loss data from the University of Maryland (available at http://earthenginepartners.appspot.com/science-2013-global-forest (accessed on 15 September 2022)). The PRODES data on deforestation rate data were obtained from INPE (http://terrabrasilis.dpi.inpe.br/ (accessed on 15 September 2022)). The original comments on these images were done by Adam Voiland (https://earthobservatory.nasa.gov/about/adam-voiland (accessed on 15 September 2022)).

On the other hand, human actions account for an unprecedented impact on the changes of the vegetal cover, at least in large areas of Brazil or Ecuador [58], reporting substantial changes on the pattern of precipitations [59] or on the complete atmospheric circulation [60]. Substantial areas of the Amazonian rain forest (Figure 4) or the “Cerrado” forest are being deforested for farming in a considerable number of sites in Brazil.

A dedicated web site hosts satellite-derived data showing the annual proportion of deforested areas in the area (PRODES deforestation rate data available at http://terrabrasilis.dpi.inpe.br/ accessed on 12 June 2022). Regarding its impact on *R. microplus*, the process of deforestation may produce (i) an increase of available habitat for the tick, (ii) an increase of the habitat fragmentation (forest/pasture/other combinations), and (iii) the availability of suitable hosts for the tick. In this sense, a relevant factor that contributes to the increase the habitat suitability for *R. microplus* is the replacement of native forest by exotic grasses used as livestock forage, which can potentially increase tick abundance not necessarily by the modification of microclimatic conditions but rather by increasing the tick-host encounter rate, because this type of pasture allows a higher stocking rate (cows/ha) than the forested areas [20,61]. The introduction of *R. microplus* in these deforested areas could happen trough cattle trading

## 5. Basics and Future of an Immunological Control of *R. microplus*

Vaccines are the most sustainable and safe alternative to the use of chemical acaricides for the control of tick infestations. The only vaccines registered and commercialized for the control of ectoparasite infestations were effective for the control of cattle tick infestations while reducing the use of acaracides (TickGard, Elders, Adelaide, Australia and Gavac, Havana, Cuba [62]). These vaccines based on the *R. microplus* Bm86 antigen were designed to develop an antibody response in vaccinated cattle affecting tick feeding, development, and reproduction [63]. In this way, vaccinations resulted in the reduction of tick populations while reducing the use of acaricides over time. These vaccines demonstrated the possibility of developing effective interventions for the immunological control of tick infestations, and further studies were conducted for the identification of more effective antigens for the control of both tick infestations and pathogen infection and transmission [64,65]. However, tick vaccines may by effective only under some conditions, and territories associated with host, environment, tick derived factors and human performance. From these studies we learned that country and host-driven approaches are needed for the effective control of cattle tick infestations [66].

We support that an effective control of cattle tick infestations can be achieved with integrated approaches combining different control interventions including a combination of synthetic and natural acaricides, environmentally sustainable management (e.g., pasture vacation and adoption of cattle with a high grade of resistance to ticks) and vaccines with country and host-driven approaches for cattle tick control. Additionally, ongoing and future research should approach other alternatives for the control of tick infestations and tick-borne diseases including (i) the application of omics technologies in combination with Big Data analytics and machine learning for the most efficient identification of tick and pathogen derived protective antigens [67]; (ii) quantum vaccinomics for the identification and combination of protective epitopes or immunological quantum to increase vaccine efficacy [68,69]; (iii) the application of new vaccine platforms such as DNA, RNA, virus-like particles, nanoparticles, multiantigenic and oral vaccine formulations for improving immune response to vaccination [70,71,72]; (iv) including in vaccine formulations other biomolecules such as glycan modifications in lipids and proteins, inactivated bacterial components and tick gut microbiota-derived bacteria to boost antigen-specific and non-specific immunity [73]; (v) paratransgenesis using gene editing by RNA interference and the latest bacterial type II Clustered Regularly Interspaced Short Palindromic Repeats and the associated protein 9 system (CRISPR-Cas9) [74], among other multidisciplinary approaches [75].

## 6. The Impact of Tick-Resistant Cattle

The control of *R. microplus* is based almost exclusively on the application of synthetic chemical acaricides, but this method, as mentioned above, has relevant drawbacks as the emergence of resistance and the accumulation of chemical residues in meat or milk. The incorporation of tick resistant cattle is a feasible tool to reduce the annual frequency of acaricide treatments. The genetic basis of host response variation to tick infestation within and between breeds is a widely known phenomenon [76,77,78,79,80,81,82,83,84,85,86]. In this sense, *Bos indicus* cattle are generally considered to be more resistant to ticks than *Bos taurus* breeds, and particular breeds from Africa (e.g., Afrikander, Nguni and N’Dama) and America (e.g., Colombian Creole breed Romosinuano, Brazilian Creole Lageano, Argentine Creole Cattle) have also shown high levels of resistance to ticks [77,78,81,83,85,86]. Because the tick burden diminishes with the increasing percentage of *B. indicus* genes in synthetic cattle breeds, the incorporation of more resistant cattle to tick infestations should be considered in areas infested with *R. microplus* where British breeds of cattle are still dominant, or cattle with a dominant composition of British genes, which are highly susceptible to *R. microplus* infestations. Furthermore, within the framework of an integrated control program, besides the increase of tick resistant genetics in a cattle herd, there is a need to evaluate the incorporation of resistant breeds that graze with highly susceptible cattle.

## 7. Conclusions

We provide a view of the several traits behind the ecology of the tick *R. microplus*, with a focus on an efficient and ecologically sustainable control. The use of vacant pasture or the timing for application of acaricides or vaccination depend mainly on the changing climate. Therefore, the development of better modeling approaches (based on physiological models) and a friendly user interface should be a pivotal point in the attempts at reducing its abundance or even its eradication. Given the tendency of the tick to develop resistance to the acaricides, the development of suitable and feasible vaccination programs should be a central aim deeply integrated with the ecological know how. A central aspect for *R microplus* tick control is the human being in charge at the cattle breeding farm. Tick control is among several routine tasks of a farm and, inevitably, rapid solutions are expected from a problem that has an ecological background and an extended tempo. Tick control solutions must be embedded in the farm’s routine, be cost-effective and ideally be able to provide, if not total, at least some relief immediately. Tick control procedures must be delivered with precise and objective guidelines and, when possible, with immediate effects. Otherwise, the indiscriminate and increasingly less efficient use of acaricides will persist with all its deleterious effects on animal, public and environmental health.

The gain of ecological knowledge aiming to the integrated control of *R. microplus* requires an interdisciplinary methodology. We can foresee a fruitful field of research using a transversal approach that incorporates environmental features and distribution modeling, rates of change of the abiotic niche as recorded by satellites, and the inclusion of cattle density (or a proxy) as further variables. The communities of possible hosts and the use of different strains of cattle in farming are important features in that transversal perspective. Such modeling approach including weather variables, habitat features, the use of soil, and the impact of deforestation has never been addressed for *R. microplus* to the best of our knowledge.

## Data Availability

Not applicable.

## References

[1] Jongejan F., Uilenberg G. (2004). The global importance of ticks. Parasitology.

[2] Canevari J.T., Mangold A.J., Guglielmone A.A., Nava S. (2017). Population dynamics of the cattle tick *Rhipicephalus (Boophilus) microplus* in a subtropical subhumid region of Argentina for use in the design of control strategies. Med. Vet. Entomol..

[3] Yessinou R.E., Akpo Y., Adoligbe C., Adinci J., Assogba M.N., Koutinhouin B., Farougou S. (2016). Resistance of tick *Rhipicephalus microplus* to acaricides and control strategies. J. Entomol. Zool. Stud..

[4] Rodriguez-Vivas R.I., Jonsson N.N., Bhushan C. (2018). Strategies for the control of *Rhipicephalus microplus* ticks in a world of conventional acaricide and macrocyclic lactone resistance. Parasitol. Res..

[5] Corson M.S., Teel P.D., Grant W.E. (2004). Microclimate influence in a physiological model of cattle-fever tick (*Boophilus* spp.) population dynamics. Ecol. Mod..

[6] Estrada-Peña A., Corson M., Venzal J.M., Mangold A.J., Guglielmone A. (2006). Changes in climate and habitat suitability for the cattle tick *Boophilus microplus* in its southern Neotropical distribution range. J. Vector Ecol..

[7] Ivancovich J.C. (1975). Bioecología de la garrapata del ganado *Boophilus microplus* (Canestrini, 1888). Rev. Inv. Agro..

[8] Ivancovich J.C., Braithwaite G.B., Barnett S.F. (1982). Comportamiento de los estados no-parasitarios de la garrapata del ganado—*Boophilus microplus* (Canestrini). I. El Colorado (provincia de Formosa). Bol. Estac. Exp. Reg. Agropecu. Pres. Roque Sáenz Peña INTA.

[9] Ivancovich J.C., Braithwaite G.B., Barnett S.F. (1984). Comportamiento de los estados no-parasitarios de la garrapata del ganado—*Boophilus microplus* (Canestrini). II. El Sombrerito (provincia de Corrientes). III. Bartolomé de las Casas (provincia de Formosa). IV. Siete diferentes localidades. Bol. Estac. Exp. Regional Agropecuaria Presidente Roque Sáenz Peña INTA.

[10] Nari. A., Cardozo H., Berdié J., Canabez F., Bawden R. (1979). Estudio preliminar sobre la ecología de *Boophilus microplus* (Can) en Uruguay. Ciclo no parasitario en un area considerada poco apta para el desarrollo. Veterinaria.

[11] Nuñez J.L., Muñoz Cobeñas M.E., Moltedo H.L. (1982). Boophilus microplus: La Garrapata Común del Ganado Vacuno.

[12] Benavides Ortiz E. (1983). Observaciones sobre la fase no parasitica del ciclo de vida de *Boop**hilus microplus* (Canestrini) en la altillanura plana Colombiana. Rev. Colomb. Entomol..

[13] Cardozo H., Nari A., Franchi M., López A., Donatti N. (1984). Estudio sobre la ecología del *Boophilus microplus* en tres áreas enzoóticas del Uruguay. Veterinaria.

[14] De La Vega R., Díaz G. (1985). Aplicación de las constantes térmicas en el control de la garrapata del ganado vacuno (*Boophilus microplus*). III. Simplificaciones en el método de estimación de los períodos de la fase no parasitaria. Rev. Salud Anim..

[15] De La Vega R., Díaz G. (1986). Aplicación de las constantes térmicas en el control de la garrapata del ganado vacuno (*Boophilus microplus*). IV. Pronóstico del inicio de la eclosión en condiciones de intemperie. Rev. Salud Anim..

[16] Gonzales J.C., da Silva N.R., Francon N., Pereira J.H. (1985). A vida libre do *Boophilus microplus* (Can. 1887). Arq. Facul. Vet. UFRGS.

[17] De Souza A.P., Gonzales J.C., Ramos C.I., Paloschi C.G., De Moraes N.A. (1988). Fase de vida livre do *Boophilus microplus* no planalto Catarinense. Pesq. Agropec. Bras..

[18] Evans D.E. (1992). Tick infestation of livestock and tick control methods in Brazil: A situation report. Int. J. Trop. Insect Sci..

[19] Brizuela C.M., Ortellano C.A., Sanchez T.I., Osorio O., Walker A.R. (1996). Formulation of integrated control of *Boophilus microplus* in Paraguay: Analysis of natural infestations. Vet. Parasitol..

[20] Nava S., Mastropaolo M., Guglielmone A.A., Mangold A.J. (2013). Effect of deforestation and introduction of exotic grasses as livestock forage on the population dynamics of the cattle tick *Rhipicephalus* (*Boophilus*) *microplus* (Acari: Ixodidae) in northern Argentina. Res. Vet. Sci..

[21] Nava S., Rossner M.V., Torrents J., Morel N., Martínez N.C., Mangold A.J., Guglielmone A.A. (2020). Management strategies to minimize the use of synthetic chemical acaricides in the control of the cattle tick *Rhipicephalus* (*Boophilus*) *microplus* (Canestrini, 1888) in an area highly favourable for its development in Argentina. Med. Vet. Entomol..

[22] Nava S., Gamietea I.J., Morel N., Guglielmone A.A., Estrada-Peña A. (2022). Assessment of habitat suitability for the cattle tick *Rhipicephalus* (*Boophilus*) *microplus* in temperate areas. Res. Vet. Sci..

[23] De Barros M.N.D.L., Riet-Correa F., Azevedo S.S., Labruna M.B. (2017). Off-host development and survival of *Rhipicephalus* (*Boophilus*) *microplus* in the Brazilian semiarid. Vet. Parasitol..

[24] Mastropaolo M., Mangold A.J., Guglielmone A.A., Nava S. (2017). Non-parasitic life cycle of the cattle tick *Rhipicephalus* (*Boophilus*) *microplus* in *Panicum maximum* pastures in northern Argentina. Res. Vet. Sci..

[25] Cruz B.C., Mendes A.F.L., Maciel W.G., Santos I.B., Gomes L.V.C., Felippelli G., Teixeira W.F.P., Ferreira L.L., Soares V.E., Lopes A.J. (2020). Biological parameters for *Rhipicephalus microplus* in the field and laboratory and estimation of its annual number of generations in a tropical region. Parasitol. Res..

[26] Vecino J.A.C., Echeverri J.A.B., Cárdenas J.A., Herrera L.A.P. (2010). Distribución de garrapatas *Rhipicephalus* (*Boophilus*) *microplus* en bovinos y fincas de Altiplano cundiboyacense (Colombia). Corpoica Cienc. Tecnol. Agropecu..

[27] Thornton P., Nelson G., Mayberry D., Herrero M. (2022). Impacts of heat stress on global cattle production during the 21st century: A modelling study. Lancet Planet. Health.

[28] Sutherst R.W., Bourne A.S. (2006). The effect of desiccation and low temperature on the viability of eggs and emerging larvae of the tick, *Rhipicephalus (Boophilus) microplus* (Canestrini) (Ixodidae). Int. J. Parasitol..

[29] Dalgliesh R.J., Stewart N.P. (1982). Some effects of time, temperature and feeding on infection rates with *Babesia bovis* and *Babesia bigemina* in *Boophilus microplus* larvae. Int. J. Parasitol..

[30] Dalgliesh R.J., Stewart N.P. (1976). Stimulation of the development of infective *Babesia bovis* (= *B. argentina*) in unfed *Boophilus microplus* larvae. Aust. Vet. J..

[31] Ouhelli H., Schein E. (1988). Effect of temperature on transovarial transmission of *Babesia bigemina* (Smith and Kilborne, 1893) in *Boophilus annulatus* (Say, 1821). Vet. Parasitol..

[32] Estrada-Peña A. (1999). Geostatistics and remote sensing using NOAA-AVHRR satellite imagery as predictive tools in tick distribution and habitat suitability estimations for *Boophilus microplus* (Acari: Ixodidae) in South America. Vet.Parasitol..

[33] Guglielmone A.A., Giorgi R., Sodiro A., Gay R., Canal A., Mangold A.J., Estrada-Peña A. (2003). Aptitud ecológica de los departamentos de Castellanos y Las Colonias, Santa Fe, para sustentar hipotéticas poblaciones de la garrapata común del vacuno, *Boophilus microplus* (Acari: Ixodidae). Rev. Inv. Agrop..

[34] Mount G.A., Haile D.G., Davey R.B., Cooksey L.M. (1991). Computer simulation of *Boophilus* cattle tick (Acari: Ixodidae) population dynamics. J. Med. Entomol..

[35] Nari-Henrioud A. (2011). Towards sustainable parasite control practices in livestock production with emphasis in Latin America. Vet. Parasitol..

[36] Nicaretta J.E., Dos Santos J.B., Couto L.F.M., Heller L.M., Cruvinel L.B., de Melo Júnior R.D., Lopes W.D.Z. (2020). Evaluation of rotational grazing as a control strategy for *Rhipicephalus microplus* in a tropical region. Res. Vet. Sci..

[37] Rossner M.V., Gomez V.D., Morel N., Nava S. (2022). Evaluación de un esquema de control integrado de *Rhipicephalus* (*Boophilus*) *microplus* en vacas preñadas y con cría en el noreste argentino. FAVE Secc. Cienc. Vet..

[38] Corson M.S., Teel P.D., Grant W.E. (2003). Simulating detection of cattle-fever tick (*Boophilus* spp.) infestations in rotational grazing systems. Ecol. Mod..

[39] Hernández-A F., Teel P.D., Corson M.S., Grant W.E. (2000). Simulation of rotational grazing to evaluate integrated pest management strategies for *Boophilus microplus* (Acari: Ixodidae) in Venezuela. Vet. Parasitol..

[40] Wang H.H., Corson M.S., Grant W.E., Teel P.D. (2017). Quantitative models of *Rhipicephalus (Boophilus)* ticks: Historical review and synthesis. Ecosphere.

[41] Kearney M. (2009). Integrating biophysical models and evolutionary theory to predict climatic impacts on species’ ranges: The dengue mosquito *Aedes aegypti* in Australia. Funct. Ecol..

[42] Porter W.P., Gates D.M. (1969). Thermodynamic equilibria of animals with environment. Ecol. Monogr..

[43] Pound J.M., George J.E., Kammlah D.M., Lohmeyer K.H., Davey R.B. (2010). Evidence for role of white-tailed deer (Artiodactyla: Cervidae) in epizootiology of cattle ticks and southern cattle ticks (Acari: Ixodidae) in reinfestations along the Texas/Mexico border in south Texas: A review and update. J. Econ. Entomol..

[44] Temeyer K.B., Chen A.C., Davey R.B., Guerrero F.D., Howell J.M., Kammlah D.M., Welch J.B. (2012). Nuevos enfoques para el control de *Rhipicephalus (Boophilus) microplus*. Rev. Mex. Cienc. Pec..

[45] Guglielmone A.A., Nava S., Robbins R.G. (2021). Neotropical Hard Ticks (Acari: Ixodida: Ixodidae).

[46] Rezende L.M., Martins M.M., Tonelotto L., Maia R.C., Rodrigues V.S., Osava C.F., Martins T.F., Labruna M.B., Szabó M.P.J. (2021). Ticks (Acari: Ixodidae) on marsh deer (*Blastocerus dichotomus*) at a conservation center: Infestation and *Rickettsia parkeri* infection dynamics along nine years. Ticks Tick-Borne Dis..

[47] Pérez de León A.A., Strickman D.A., Knowles D.P., Fish D., Thacker E., de la Fuente J., Pound J.M. (2010). One Health approach to identify research needs in bovine and human babesioses: Workshop report. Parasites Vectors.

[48] Showler A.T., Pérez de León A. (2020). Landscape ecology of *Rhipicephalus (Boophilus) microplus* (Ixodida: Ixodidae) outbreaks in the South Texas coastal plain wildlife corridor including man-made barriers. Environ. Entomol..

[49] Szabó M.P.J., Labruna M.B., Pereira Campos M., Duarte J.M.B. (2003). Ticks (Acari: Ixodidae) on wild marsh-deer (*Blastocerus dichotomus*) from Southeast of Brazil: Infestations prior and after habitat loss. J. Med. Entomol..

[50] Ojeda-Chi M.M., Rodriguez-Vivas R.I., Esteve-Gasent M.D., Pérez de León A., Modarelli J.J., Villegas-Perez S. (2019). Molecular detection of rickettsial tick-borne agents in white-tailed deer (*Odocoileus virginianus yucatanensis*), mazama deer (*Mazama temama*), and the ticks they host in Yucatan, Mexico. Ticks Tick-Borne Dis..

[51] Cançado P.H.D., Zucco C.A., Piranda E.M., Faccini J.L.H., Mourão G.M. (2009). *Rhipicephalus* (*Boophilus*) *microplus* (Acari: Ixodidae) as a parasite of pampas deer (*Ozoctoceros bezoarticus*) and cattle in Brazil’s Central Pantanal. Rev. Bras. Parasitol. Vet..

[52] Silveira J.A.G., Rabelo E.M.L., Lacerda A.C.R., Borges P.A.L., Tomás W.M., Pellegrin A.O., Tomich R.G., Ribeiro M.F. (2013). Molecular detection and identification of hemoparasites in pampas deer (*Ozotoceros bezoarticus* Linnaeus, 1758) from the Pantanal Brazil. Ticks Tick-Borne Dis..

[53] Benitez D., Cetrá B., Florin-Christensen M. (2012). *Rhipicephalus* (*Boophilus*) *microplus* ticks can complete their life cycle on the water buffalo (*Bubalus bubalis*). J. Buffalo Sci..

[54] Lohmeyer K.H., May M.A., Thomas D.B., Pérez de León A. (2018). Implication of nilgai antelope (Artiodactyla: Bovidae) in reinfestations of *Rhipicephalus (Boophilus) microplus* (Acari: Ixodidae) in South Texas: A review and update. J. Med. Entomol..

[55] Estrada-Peña A. (2002). Understanding the relationships between landscape connectivity and abundance of *Ixodes ricinus* ticks. Exp. Appl. Acarol..

[56] Estrada-Peña A. (2003). The relationships between habitat topology, critical scales of connectivity and tick abundance *Ixodes ricinus* in a heterogeneous landscape in northern Spain. Ecography.

[57] Estrada-Peña A., Venzal J.M. (2006). High-resolution predictive mapping for *Boophilus annulatus* and *B. microplus* (Acari: Ixodidae) in Mexico and Southern Texas. Vet. Parasitol..

[58] Costa M.H., Pires G.F. (2010). Effects of Amazon and Central Brazil deforestation scenarios on the duration of the dry season in the arc of deforestation. Int. J. Climatol..

[59] Medvigy D., Walko R.L., Avissar R. (2011). Effects of deforestation on spatiotemporal distributions of precipitation in South America. J. Clim..

[60] Ruiz-Vásquez M., Arias P.A., Martínez J.A., Espinoza J.C. (2020). Effects of Amazon basin deforestation on regional atmospheric circulation and water vapor transport towards tropical South America. Clim. Dyn..

[61] Guglielmone A.A. (1992). The level of infestation with the vector of cattle babesiosis in Argentina. Mem. Inst. Oswaldo Cruz.

[62] De la Fuente J., Almazán C., Canales M., Pérez de la Lastra J.M., Kocan K.M., Willadsen P. (2007). A ten-year review of commercial vaccine performance for control of tick infestations on cattle. Anim. Health Res. Rev..

[63] De la Fuente J., Rodríguez M., Redondo M., Montero C., García-García J.C., Méndez L., Serrano E., Valdés M., Enriquez A., Canales M. (1998). Field studies and cost-effectiveness analysis of vaccination with Gavac against the cattle tick *Boophilus microplus*. Vaccine.

[64] De la Fuente J., Kocan K.M. (2003). Advances in the identification and characterization of protective antigens for recombinant vaccines against tick infestations. Expert Rev. Vaccines.

[65] De la Fuente J., Contreras M. (2015). Tick vaccines: Current status and future directions. Expert Rev. Vaccines.

[66] De la Fuente J., Contreras M., Kasaija P.D., Gortazar C., Ruiz-Fons J.F., Mateo R., Kabi F. (2019). Towards a multidisciplinary approach to improve cattle health and production in Uganda. Vaccines.

[67] De La Fuente J., Villar M., Estrada-Peña A., Olivas J.A. (2018). High throughput discovery and characterization of tick and pathogen vaccine protective antigens using vaccinomics with intelligent Big Data analytic techniques. Expert Rev. Vaccines.

[68] De la Fuente J., Contreras M. (2021). Vaccinomics: A future avenue for vaccine development against emerging pathogens. Expert Rev. Vaccines.

[69] Contreras M., Artigas-Jerónimo S., Pastor Comín J.J., de la Fuente J. (2022). A Quantum Vaccinomics Approach Based on Protein-Protein Interactions. Methods Mol. Biol..

[70] Bogani G., Raspagliesi F., Ditto A., de la Fuente J. (2020). The Adoption of Viral Capsid-Derived Virus-Like Particles (VLPs) for Disease Prevention and Treatments. Vaccines.

[71] Kasaija P.D., Contreras M., Kabi F., Mugerwa S., Garrido J.M., Gortazar C., de la Fuente J. (2022). Oral vaccine formulation combining tick Subolesin with heat inactivated mycobacteria provides control of cross-species cattle tick infestations. Vaccine.

[72] De la Fuente J., Contreras M. (2022). Additional considerations for anti-tick vaccine research. Expert Rev. Vaccines.

[73] Mateos-Hernández L., Obregón D., Maye J., Borneres J., Versille N., de la Fuente J., Estrada-Peña A., Hodžić A., Šimo L., Cabezas-Cruz A. (2020). Anti-Tick microbiota vaccine impacts *Ixodes ricinus* performance during feeding. Vaccines.

[74] Sharma A., Pham M.N., Reyes J.B., Chana R., Yim W.C., Heu C.C., Kim D., Chaverra-Rodriguez D., Rasgon J.L., Harrell R.A. (2022). Cas9-mediated gene editing in the black-legged tick, *Ixodes scapularis*, by embryo injection and ReMOT Control. iScience.

[75] De la Fuente J. (2021). Translational biotechnology for the control of ticks and tick-borne diseases. Ticks Tick-Borne Dis..

[76] Bennett G.F., Wharton R.H. (1968). Variability of host resistance to cattle tick. Proceedings of the Ecological Society of Australia.

[77] Cardoso C.P., Silva B.F., Gonçalves D.S., Tagliari N.J., Saito M.E., Amarante A.F.T. (2014). Resistência contra ectoparasitas em bovinos da raça Crioula Lageana e meiosangue Angus avaliada em condições naturais. Pesqui. Vet. Bras..

[78] Fivaz B.H., de Waal D.T., Lander K. (1992). Indigenous and crossbred cattle. A comparison of resistance to ticks and implications for their strategic control in Zimbabwe. Trop. Anim. Health Prod..

[79] Francis J. (1966). 1966. Resistance of Zebu and other cattle to tick infestation and Babesiosis with special reference to Australia: An historical review. Br. Vet. J..

[80] Frisch J., O’Neill C. (1998). Comparative evaluation of beef cattle breeds of African, European and Indian origins. 2. Resistance to cattle ticks and gastrointestinal nematodes. Anim. Sci..

[81] Guglielmone A.A. (1990). Parasitismo natural por *Boophilus microplus* de bovinos Hereford, Criolla, Nelore y cruzas Hereford x Nelore. Rev. Med. Vet..

[82] Jonsson N.N., Piper E.K., Constantinoiu C.C. (2014). Host resistance in cattle to infestation with the cattle tick *Rhipicephalus microplus*. Parasite Immunol. Spec. Issue Immunol. Ectoparasite Infect..

[83] Burrow H.M., Mans B.J., Cardoso F.F., Birkett M.A., Kotze A.C., Hayes B.J., Mapholi N., Dzama K., Marufu M.C., Githaka N.W. (2019). Towards a new phenotype for tick resistance in beef and dairy cattle: A review. Anim. Prod. Sci..

[84] Utech K.B.W., Wharton R.H., Kerr J.D. (1978). Resistance to *Boophilus microplus* (Canestrini) in different breeds of cattle. Aust. J. Agric. Res..

[85] Wilkinson P.R. (1955). Observations on infestations of undipped cattle of British breeds with the cattle tick, *Boophilus microplus* (Canestrini). Aust. J. Agric. Res..

[86] Scholtz M.M., Spickett A.M., Lombard P.E., Enslin C.B. (1991). The effect of tick infestation on the productivity of cows of three breeds of cattle. Onderstepoort J. Vet. Res..

